# CU06-1004 Alleviates Experimental Colitis by Modulating Colonic Vessel Dysfunction

**DOI:** 10.3389/fphar.2020.571266

**Published:** 2020-09-15

**Authors:** Ye-Seul Kim, Haiying Zhang, Sunghye Lee, Songyi Park, Minyoung Noh, Young-Myeong Kim, Young-Guen Kwon

**Affiliations:** ^1^Department of Biochemistry, College of Life Science and Biotechnology, Yonsei University, Seoul, South Korea; ^2^R&D Department, Curacle Co. Ltd, Seongnam-si, South Korea; ^3^Vascular System Research Center, Kangwon National University, Chuncheon, South Korea

**Keywords:** CU06-1004, inflammatory bowel disease, ulcerative colitis, endothelium, endothelial dysfunction, colonic vessel, epithelium, epithelial barrier

## Abstract

Inflammatory bowel disease is an autoimmune disease that causes chronic inflammation of the gastrointestinal tract. Endothelial dysfunction, defined by a reduced endothelial barrier and an increase in the expression of adhesion molecules, is part of the pathology of inflammatory bowel disease. In this study, we assessed the therapeutic effect of CU06-1004, an endothelial dysfunction blocker that reduces vascular hyperpermeability and inflammation in a mouse model of colitis. Acute colitis was induced in mice using 3% (w/v) dextran sodium sulfate added to their drinking water for 7 days. Twenty-four hours after the addition of dextran sodium sulfate, either mesalazine or CU06-1004 was administered orally each day. Administration of CU06-1004 significantly reduced the clinical manifestations (weight loss, diarrhea, and bloody stool) and histological changes (epithelium loss, inflammatory cell infiltration, and crypt destruction) induced by dextran sodium sulfate. Proinflammatory cytokines were also reduced, indicating that inflammation was ameliorated. From a vascular perspective, CU06-1004 reduced interrupted and tortuous vessels, enhanced junction protein expression, and reduced inflammatory adhesion molecules, indicating a broad improvement of endothelial dysfunction. Endothelial protection induced epithelial barrier restoration and decreased epithelial inflammation. Blocking endothelial dysfunction with CU06-1004 significantly ameliorated the progression of inflammatory bowel disease. Therefore, CU06-1004 may represent a potential therapeutic agent for the treatment of inflammatory bowel disease as well as other inflammatory diseases.

## Introduction

Inflammatory bowel disease (IBD) is a chronic gastrointestinal disorder associated with changes in mucosal immunity caused by a loss of intestinal homeostasis. It is characterized by abdominal pain, weight loss, diarrhea, and bloody stool (Khor et al., 2011; Grinspan and Kornbluth, 2015). The dysregulated intestinal inflammation associated with IBD can also induce complications such as fistulas, stenosis, and abscesses (Blumberg, 2009). Furthermore, IBD patients have a higher risk of developing colorectal cancer and colitis-associated cancer (Mattar et al., 2011).

IBD consists of Crohn’s disease (CD) and ulcerative colitis (UC). These diseases have different but overlapping features. CD affects the entire gastrointestinal tract, but the terminal ileum and colon are most commonly affected. Moreover, it is characterized by an upregulation of interferon-γ, which has a pivotal role in Th1-type responses. On the other hand, UC inflammation occurs typically in the colon and rectum, and is associated with a Th2-type response featuring interleukin-4 and interleukin-13 secretions and activated natural killer T (NK-T) cells. NK-T cells are highly cytotoxic to epithelial cells, aggravating UC, by reducing the epithelial barrier (Brown and Mayer, 2007; Dharmani and Chadee, 2008). CD and UC also have common features: imbalances between proinflammatory and antiinflammatory cytokines. Proinflammatory mediators such as interleukin-1beta (IL-1β), interleukin-6 (IL-6), and tumor necrosis factor-alpha (TNF-α) are found at high levels in the colonic mucosa. These mediators are produced by innate immune cells such as macrophages and induce cytotoxicity, apoptosis, and an acute-phase response (Papadakis and Targan, 2000). However, the antiinflammatory cytokine levels of both interleukin-4 and interleukin-10 are decreased. Interestingly, IBD patients (both CD and UC) present with lower intra-luminal pH due to inflammation compared with healthy people. Leukocyte infiltration of the gut and the concomitant increase in metabolic by-product levels makes the gut more acidic by impairing the cellular waste-removal process (Lardner, 2001; Nugent et al., 2001; Barkas et al., 2013).

In the intestine, blood vessels have many roles, such as supporting oxygen and nutrient exchange, maintaining osmotic balance, and allowing leukocyte migration in the extracellular compartment. During IBD, these blood vessels undergo fast and remarkable changes by a process known as immune-derived angiogenesis. Due to this angiogenesis, the total vessel surface area increases, which provides an opportunity for immune cell extravasation. In addition, endothelial dysfunction includes increased vascular permeability increases, and upregulation of adhesion molecules occurs not only in these new blood vessels in IBD patients, but also in their existing intestinal vasculature. This increased vascular permeability reduces overall barrier function and allows for luminal bacteria-leukocyte interactions, which cause intestinal hypoxia and increased expression of free radicals and inflammatory cytokines, and lead to intestinal epithelial cell damage. Given that the upregulation of these adhesion molecules is linked to disease severity (Cromer et al., 2011; Alkim et al., 2015; Gravina et al., 2018; Sun et al., 2018), regulating these intestinal blood vessel changes is likely to be important for the prevention and treatment of IBD.

No studies have examined the endothelial dysfunction blocker, CU06-1004, for therapeutic efficacy in the inflammatory diseases such as IBD. Therefore, we assessed its therapeutic potential using a dextran sodium sulfate (DSS)-induced colitis model in the mouse. We previously demonstrated that CU06-1004 enhances endothelial cell survival and integrity *via* the upregulation of cyclic adenosine monophosphate levels and Rac signal activation that leads to cortical actin ring formation. CU06-1004 blocks endothelial hyperpermeability and the loss of junction proteins induced by multiple factors such as vascular endothelial growth factor, IL-1β, and histamine. It also inhibits the expression of inflammatory adhesion molecules such as ICAM-1 and VCAM-1 by nuclear factor-κB inhibition. In other pathologies such as cancer, diabetic retinopathy, and mouse models of cerebral ischemia, the therapeutic influence of CU06-1004 has been *via* the inhibition of vascular hyperpermeability and inflammation (Maharjan et al., 2013; Agrawal et al., 2014; Lee et al., 2014; Batbold et al., 2016; Zhang et al., 2017). Here, we demonstrate that oral administration of CU06-1004 ameliorated the macroscopic pathology of the colon as well as histological changes and inflammation. These therapeutic effects correlate with reductions in endothelial abnormalities and inflammatory cytokine expression, and result in the preservation of the epithelial barrier, which blocks further progression of IBD.

## Materials and Methods

### Test Drug

CU06-1004, previously described as sac-1004, was synthesized as described previously.19 Briefly, CU06-1004 was tetrahydropyran-deprotected and subsequently glycosylated with 4, 6-di-O-acetyl-2,3-didieoxyhex-2-enopyran in the presence of acid. Mesalazine was purchased from Sigma-Aldrich, St. Louis, MO, USA. CU06-1004 and mesalazine were dissolved in olive oil. Since CU06-1004 was observed to be most effective at a dose of 10 mg/kg, that was the dose used in all experiments (**Figure S1** in **Supplementary Material**).

### Mice

Male C57BL/6J mice (8–10 weeks) were obtained from DBL (Eumsung, Korea) and acclimated for one week under controlled conditions (12 h/12 h dark-light cycle, 22°C ± 1°C, 50%–60% humidity). All mice had access to sterilized tap water and standard mouse chow *ad libitum*. Mice maintained under pathogen-free conditions at Yonsei University. All animal experiments were conducted under the institutional guidelines established for the Animal Core Facility at Yonsei University of Medicine with approval of the institutional care and use committee.

### DSS-Induced Colitis

Mice were randomly assigned to four groups numbering 4–6 mice: control, DSS, DSS+mesalazine, and DSS+1004. The control group received distilled water (no colitis induction). Mice in the other groups received a 3% aqueous solution (w/v) of DSS (M.W. = 36,000–50,000 Da) (MP Biochemicals, Solon, OH, USA) *ad libitum* for 7 days, replaced every other day (Kim et al., 2012). Control- and DSS-group mice received vehicle (olive oil) by oral gavage 24 h after DSS was provided. The DSS+1004 group received oral 1,004 (10 mg/kg body weight, dissolved in olive oil), and the DSS+mesalazine group received oral mesalazine (Sigma-Aldrich; 100 mg/kg body weight, dissolved in olive oil). Each experiment was repeated four times.

### Assessment of Disease Severity

pt?>In each repeat, mice were examined daily for body weight, stool consistency, and fecal blood. These three factors were used to determine the additive disease activity index (DAI) score. Scores were determined by: (1) weight loss (0 = <1%, 1 = 1%–5%, 2 = 5%–10%, 3 = 10%–20%, 4 = >20%); (2) stool consistency (0 = normal, 2 = loose stool, 4 = diarrhea); and (3) fecal blood (0 = no blood, 2 = red, 4 = black) (Cooper et al., 1993). On day 7, mice were euthanized, and both colon length and weight were measured to calculate the edema rate (length/weight ratio). Data were expressed as the sum of mice across the four repeat experiments (weight loss was calculated relative to day 0 weight for each mouse. Stool consistency and fecal blood scores were examined quantitatively, not as relative measures. Therefore, all repeats of experiments were pooled and presented together). And the presented image was chosen in the same experiment, and has close to the median value.

### Histopathological Assessment

After each mouse euthanized, its colon was removed and washed with cold phosphate-buffered saline (PBS). The distal colon was then fixed for 48 h in cold 4% paraformaldehyde (PFA), embedded in paraffin, and sectioned at 4–6 μm. Sections were stained with hematoxylin and eosin (H&E) and observed under a phase-contrast microscope (100× magnification, Nikon, Japan). Epithelial loss, crypt disruption, and inflammatory cell infiltration were each graded separately: (0 = no change, 1 = localized and mild, 2 = localized and moderate, 3 = extensive and moderate, and 4 = extensive and severe). The three grades were then combined to obtain a total histological score (Nishiyama et al., 2012). Staining was repeated for each experimental repeat. Data were expressed as the sum of mice across the four repeat experiments. Images were chosen from an experiment that was close to the median value for that group.

### Immunofluorescence Analysis

Distal colon sections (from the same sample used for H&E staining) were deparaffinized in xylene and then rehydrated with an alcohol gradient. For antigen retrieval (heat-induced epitope retrieval), samples were heated in 10 mM sodium citrate buffer using a microwave oven and then allowed to cool to room temperature. Sections were treated with 3% hydrogen peroxide to block endogenous peroxidase activity and permeabilized with 0.4% Triton X-100 in PBS. Sections were blocked with Protein Block solution (Dako, Santa Cruz, CA, USA) for 1 h and incubated with the following primary antibodies overnight at 4°C: anti-goat CD31 (R&D Systems, 1:200), anti-rabbit ZO-1 (Invitrogen, 1:100), anti-rabbit occludin (Invitrogen, 1:200), anti-rabbit claudin-5 (Invitrogen, 1:200), anti-rabbit VE-cadherin (Invitrogen, 1:200). Sections then incubated with appropriate secondary antibodies labeled with Alexa Fluor 488 or Alexa Fluor 594. Before imaging, nuclei stained using 4′,6-diamidino-2-phenylindole (DAPI). Images captured using a confocal microscope (Carl Zeiss). Staining was repeated for each experimental repeat. Data were expressed for single repeat of experiments. Images were chosen from an experiment that was close to the median value for that group.

### Colon Whole-Mount Immunostaining

Colon whole-mount processing was performed as previously described (Bernier-Latmani and Petrova, 2016). Taking 7 days to complete. Briefly, an anesthetized mouse was perfused with 1× PBS and 1% (w/v) PFA by cardiac puncture. The colon was harvested, cut longitudinally, washed with ice-cold 1× PBS, and pinned onto a silicon plate lumen-side up. Colon samples were then post-fixed in 4% PFA overnight. On day 2, samples were washed with ice-cold PBS, incubated with 10% sucrose solution for 3 h at 4°C on an orbital shaker, and then incubated overnight in a 20% sucrose-glycerol solution at 4°C on an orbital shaker. On day 3, after washing with ice-cold PBS, the colon was cut into fragments measuring 0.5–1 cm in length and immersed in a blocking solution for 2 h at 4°C on an orbital shaker. Primary antibody treatment; CD31 (BD bioscience, 1:500) in blocking solution was performed overnight at 4°C on an orbital shaker. On day 4, segments were washed with ice-cold 0.3% PBS-Tween (PBS-T) and then incubated with secondary antibody (Alexa Fluor 594) in blocking solution overnight at 4°C on an orbital shaker. On day 5, segments were washed in ice-cold 0.3% PBS-T and then immersed in 4% PFA for two days at 4°C on an orbital shaker. On day 7, after washing with ice-cold PBS, segments were treated with FocusClear solution (CelExplorer labs, HSINCHU, TAIWAN) for 20 min. The FocusClear solution was then removed, and the segments were mounted with ProLong Gold (Life Technologies, CA, USA). Z-stack images were acquired using a confocal microscope (Carl Zeiss). Whole-mount staining was done in one of the four experimental repeats. Images were chosen that reflect the median value for each group.

### RNA Isolation, Purification, and Quantitative Real-Time Polymerase Chain Reaction

Total RNA was extracted from colon samples using Easy-Blue reagent (Intron, Sungnam, Korea) according to the manufacturer’s instructions. RNA was purified by lithium chloride and sodium acetate precipitation to remove any remaining DSS (a possible polymerase inhibitor). The RNA solution was mixed with 0.1 volume of 8 M LiCl (Sigma-Aldrich), incubated on ice for 2 h, and then centrifuged at 14,000*g* for 30 min, at 4°C. The supernatant discarded and the pellet dissolved in diethyl pyrocarbonate-treated water. This procedure repeated once and then 0.1 volume of 3 M sodium acetate (Sigma-Aldrich) and two volumes of pre-chilled 100% ethanol were added. After a 30-min incubation, the RNA solution was centrifuged at 14,000*g* for 10 min at 4°C. The RNA pellet was then washed with pre-chilled 70% ethanol. The supernatant discarded, and the dried RNA pellet was dissolved in a volume smaller (or equal to) the initial volume (Viennois et al., 2018). RNA concentrations were determined using a Nanodrop instrument (Thermo Fisher Scientific). Reverse transcription of RNA (2 μg) into cDNA performed using reverse transcriptase (Promega, Madison, WI, USA) according to the manufacturer’s instructions. Quantitative real-time polymerase chain reaction (qRT-PCR) performed using a SYBR Green master mix (Thermoscientific, Waltham, MA, USA) with an initial denaturation step at 95°C for 10 min, followed by 40 cycles of 95°C for 15 s, 60°C for 30 s, 70°C for 30 s, and 60°C for 15 s. Primer sequences are presented in the following **Table 1**. mRNA expression levels for each target gene were normalized to glyceraldehyde 3-phosphate dehydrogenase levels. Relative expression was determined using the 2^-ΔΔCt^ (fold change) method based on control mouse RNA as a reference. qRT-PCR was repeated twice in each of the four experimental repeats.

**Table 1:** Primer list.

gene	Forward	Reverse
IL-1B	TGGACCTTCCAGGATGAGGACA	GTTCATCTCGGAGCCTGTAGTG
TNF-a	GGTGCCTATGTCTCAGCCTCTT	GCCATAGAACTGATGAGAGGGAG
IL-6	TACCACTTCACAAGTCGGAGGC	CTGCAAGTGCATCATCGTTGTTC
E-selectin	GGACACCACAAATCCCAGTCTG	TCGCAGGAGAACTCACAACTGG
P-selectin	AAGATGCCTGGCTACTGGACAC	CAAGAGGCTGAACGCAGGTCAT
VCAM1	GCTATGAGGATGGAAGACTCTGG	ACTTGTGCAGCCACCTGAGATC
ICAM1	AAACCAGACCCTGGAACTGCAC	GCCTGGCATTTCAGAGTCTGCT
CXCL12	GGAGGATAGATGTGCTCTGGAAC	AGTGAGGATGGAGACCGTGGTG
CXCR4	GACTGGCATAGTCGGCAATGGA	CAAAGAGGAGGTCAGCCACTGA
HO-1	CACTCTGGAGATGACACCTGAG	GTGTTCCTCTGTCAGCATCACC
iNOS	GAGACAGGGAAGTCTGAAGCAC	CCAGCAGTAGTTGCTCCTCTTC
FLT1	TGGATGAGCAGTGTGAACGGCT	GCCAAATGCAGAGGCTTGAACG

### Evan’s Blue Assay

After the DAI and drug supply (at day 7), 0.5% w/v Evan’s blue was administered intravenously. Two hours later, the mice were euthanized and perfused with sterile PBS. For this experiment, we administered 4 μl of 0.5% w/v Evan’s blue per 1g body weight. After sacrifice, the colon was harvested, weighed, and dye was extracted with formamide (4 μl formamide per 1 mg of colon tissue) for 48 h at 60 . Then, we measured absorbance at 620 nm. These data were presented as the fold change in OD620 relative to the control group.

### Statistical Analyses

All results are presented as means ± standard errors of the mean (SEM). An analysis of variance was used for comparisons between groups. The difference of the means among groups was statistically analyzed by one-way or two-way analysis of variance (ANOVA) with followed by turkey’s test. A P-value of <0.05 was considered statistically significant. Analyses performed using GraphPad Prism version 7.0 (GraphPad Software, San Diego, CA, USA).

## Results

### CU06-1004 Reduced the Severity of DSS-Induced Colitis

**Figure 1:**
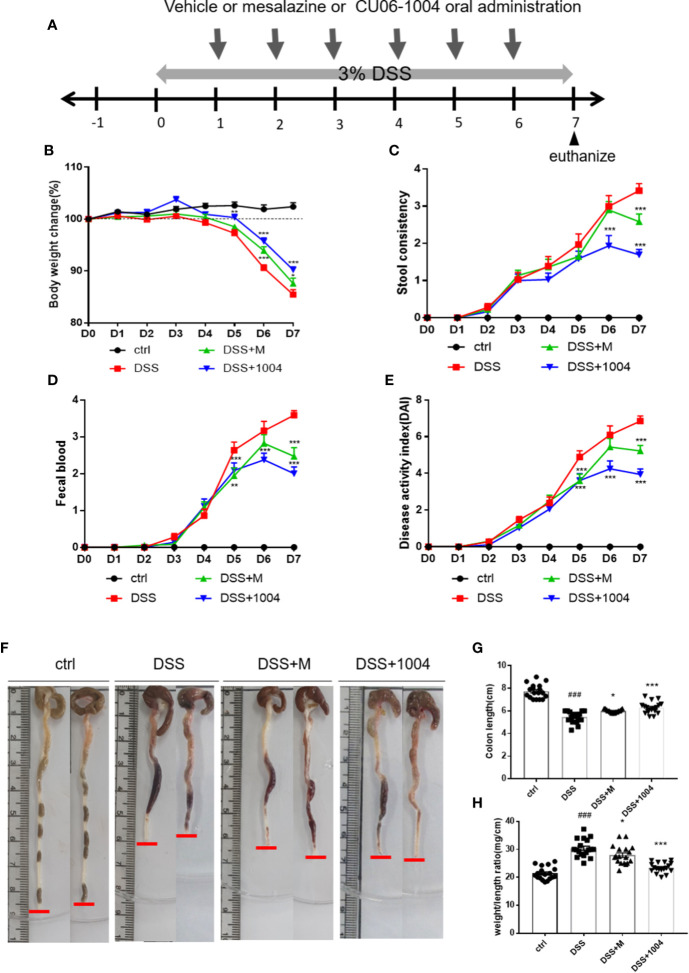
CU06-1004 treatment attenuated the clinical features of dextran sodium sulfate (DSS)-induced colitis in mice. Mice divided into four groups; control (ctrl.), vehicle (DSS), Mesalazine (DSS+M), and CU06-1004 (DSS+1004). The control (normal) group received water and the other groups received Dextran Sodium Sulfate. Mice were administered 3% DSS *ad libitum *for 7 days, and vehicle or Mesalazine or CU06-1004 treated 24 h after DSS administration started. Mesalazine (an FDA-approved TNFα inhibitor) administered at 100 mg/kg per day, and CU06-1004 at 10 mg/kg, for 6 days **(A)**. Body weight change (%) **(B)**, diarrhea **(C)**, and fecal blood scores **(D)** were determined each day, and their sums determined a *disease activity index* (DAI) score **(E)**. Representative images of removed mouse colon **(F)**, colon length measurement **(G)**, and weight-length ratios **(H)**. ^###^P < 0.001 versus the control group. *P < 0.05, **P < 0.01, ***P < 0.001 versus the DSS group. (n=18-20 per group).

The DSS mouse model was used to induce severe mucosal inflammation and colitis, a model for UC. Its pathophysiological and morphological characteristics are considered similar to UC (Okayasu et al., 1990; Whittem et al., 2010). Mesalazine (5-aminosalicylic acid; 5-ASA), a Food and Drug Administration (FDA)-approved drug to treat UC, was used as a positive control treatment. The experimental scheme was shown in **Figure 1A**. The DAI, an indicator of UC severity in mice, was used for the daily assessment of body weight, stool consistency, and fecal blood. Body weight reductions in all DSS-treated groups began on day 4, with the other factors indicating severity on day 3. In the CU06-1004-treated group, this body weight reduction significantly prevented from day 4. Weight loss also blocked in the mesalazine-treated group (**Figure 1B**). Changes in stool consistency and fecal blood were also ameliorated in both of these groups (**Figures 1C, D**), leading to significantly decreased DAI scores in both CU06-1004- and mesalazine-treated mice (**Figure 1E**). Colon shortening is another indicator of colorectal inflammation, and the average colon length of the control group was 7.6 cm, but the average length in DSS-groups reduced to 5.43 cm. Average colon lengths from the mesalazine- and CU06-1004-treated groups were 5.93 cm and 6.14 cm, respectively (**Figures 1F, G**). An additional indicator of inflammation, the colon weight-length ratio (mg/cm), decreased by approximately 10% and 18% in the drug-treated groups (**Figure 1H**).

### CU06-1004 Treatment Attenuated Histological Damage and Leukocyte Infiltration

**Figure 2:**
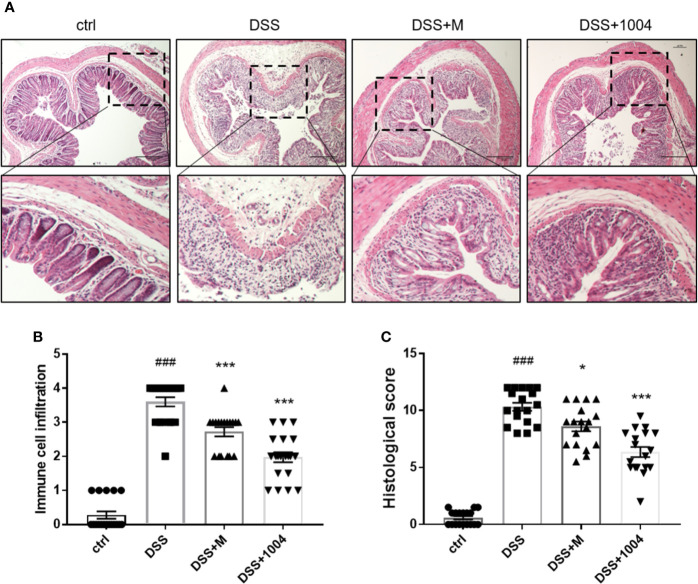
CU06-1004 prevented tissue damage and the infiltration of inflammatory cells into the colon. Colons were obtained 7 days after dextran sodium sulfate (DSS) administration and then sectioned and stained with H&E. Magnification 100×, scale bar: 20μm **(A)**. Histopathological scores were then assessed **(B, C)**. ^###^P < 0.001 versus the control group. *P < 0.05, ***P < 0.001 versus the DSS group. (n=18–20 per group).

DSS treatment destroyed intestinal epithelial cells and crypt structure and induced inflammatory cell infiltration. Histological analyses of the distal colon performed using H&E-stained sections. Control-group mice exhibited an intact epithelial cell layer and full-crypt structure without any leukocyte infiltration. In contrast, DSS-administered mice showed epithelial cell and crypt damage with high levels of leukocyte infiltration. Tissues from both mesalazine- and CU06-1004-treated mice showed preserved colon histology, with reduced inflammation, compared with the DSS-only group (**Figures 2A, B**). The histological scores also showed that DSS induced histological defects and that these reduced by mesalazine and CU06-1004 treatments (**Figure 2C**).

### CU06-1004 Decreased the Level of Proinflammatory Cytokines in the Colitis-Induced Colon

**Figure 3:**
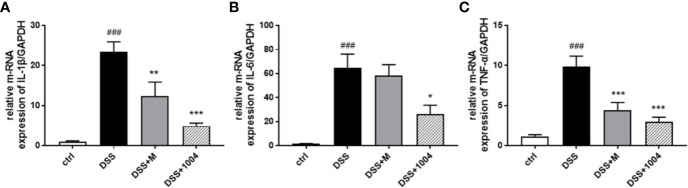
The effect of CU06-1004 on the expression of inflammatory biomarkers. Quantitative real-time polymerase chain reaction (qRT-PCR) performed using gut homogenates to detect proinflammatory cytokine expression levels. IL-1β **(A)**, IL-6 **(B)**, and TNF-α **(C)** ###P < 0.001, versus the control group. *P < 0.05, **P < 0.01, ***P < 0.001 versus the DSS group. (n=4–5 per group).

Persistent inflammation is important for the development of UC. IL-1β, IL-6, and TNF-α all play critical roles in IBD progression. Therefore, we investigated the effects of CU06-1004 on the expression of these proinflammatory cytokines. Compared to control-group mice, the levels of IL-1β, IL-6, and TNF-α increased in DSS-group mice. However, mesalazine- and CU06-1004-group mice showed significantly reduced cytokine levels (**Figures 3A–C**). These results suggest that CU06-1004 has an antiinflammatory effect to prevent DSS-induced colitis.

### CU06-1004 Maintained Blood Vessel Integrity in the Colon

**Figure 4:**
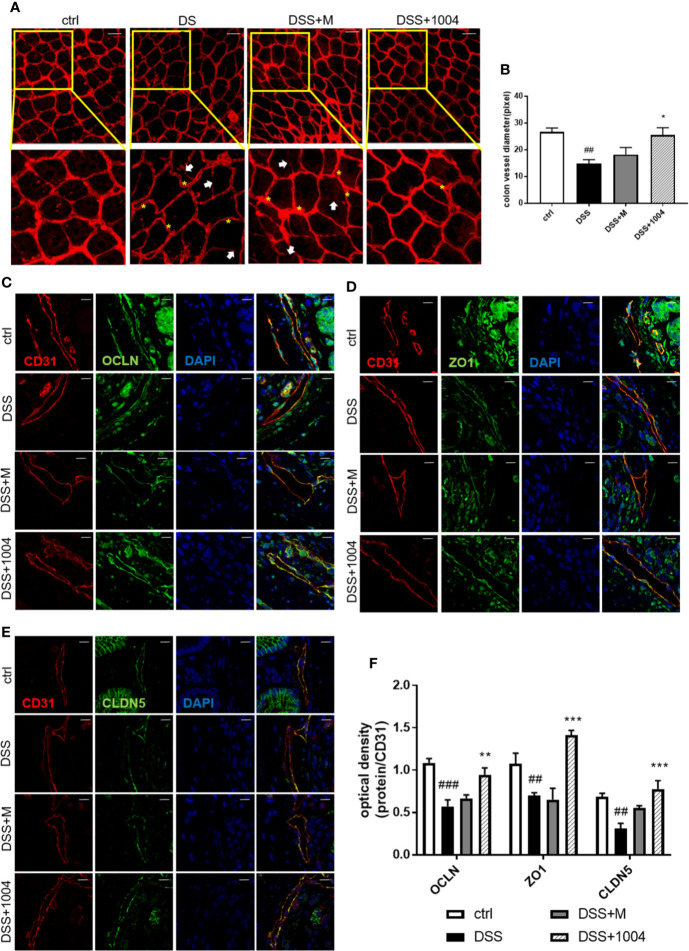
CU06-1004 attenuated vascular abnormalities and barrier destruction. Whole-colon immunofluorescence staining for CD31 (red), with an interrupted vessel (white arrow), and tortuous vessel (yellow asterisk) Magnification 200×, scale bar: 50 μm **(A)**. Vessel diameters measured by counting the number of pixels on vascular sections viewed by confocal microscopy (n=4 per group) **(B)**. Immunofluorescence staining of colon sections for CD31 (red) and the tight junction proteins (green) occludin **(C)**, Zonula occludens-1 **(D)**, and claudin-5 **(E)**. Sections were counterstained with 4′,6-diamidino-2-phenylindole (DAPI) (blue nuclei). CD31 and tight junction co-expression were quantified by optical density **(F)**. (n=4–5 per group). The coexpression of junction proteins and vascular markers represent the colon’s vascular barrier. Magnification 1,000×, scale bar: 10 μm, ^##^P < 0.01, ^###^P < 0.001 versus the control group. *P < 0.05, **P < 0.01, ***P < 0.001 versus the dextran sodium sulfate (DSS) group.

During the development of UC, blood vessels in the colon lose their normal integrity and appearance. During colitis, there is loss of vascular integrity. Indeed, the increase in colonic vascular permeability precedes the clinical symptoms and accelerates the development of colitis (Tolstanova et al., 2012). Colonic vessels become interrupted, tortuous, and have decreased diameters during colitis (Chidlow et al., 2007). While the DSS-administered group had increased leakage of vascular Evan’s blue, the CU06-1004-treated group exhibited markedly decreased residual Evan’s blue, suggesting that CU06-1004 reduced colonic vascular permeability (**Figure S2** in **Supplementary Material**). Blood vessels in the DSS and mesalazine-administered group colonic vessel were interrupted (**Figure 4A**, white arrow), and were tortuous (yellow asterisk). However, blood vessels in CU06-1004-treated mice were less interrupted and less tortuous than the other group (**Figure 4A**). The CU06-1004-treated group also had larger vessel diameters than the other group of mice (**Figure 4B**). During UC, vascular permeability in the colon increases (i.e., a loss of integrity), causing bleeding and inflammation (Saijo et al., 2015), and tight junctions play a pivotal role in maintaining vascular integrity (Erickson et al., 2007; Edelblum and Turner, 2009). As CU06-1004 has been reported to block such leakage, we investigated whether its influence on these junctions. In the colon, tight junction proteins are expressed not only in the endothelium but also in the epithelium. As our focus was vascular, colon samples were double-stained for tight junction proteins and an endothelial cell marker (CD31) to exclude the epithelium. Occludin (OCLN), Zonula occludens-1 (ZO-1), and claudin-5 (CLDN-5) reported representing tight junction proteins that maintain vascular junctions. Expressions of OCLN, ZO-1, and CLDN-5 in DSS-group mice decreased. The colonic vessel junctions in the mesalazine group were slightly increased (no significance), and there was no apparent difference compared to DSS-treated mice. However, tight junction coverage was enhanced by approximately 53% in CU06-1004-treated mice versus DSS mice (**Figures 4C–F**). This strengthening of the vascular junctions also caused a reduction in hypoxia (**Figure S3A** in **Supplementary Material**) and pathological changes (**Figures S3B, C** in **Supplementary Material**), which may also prevent an increase in colitis severity.

### The CU06-1004 Antiinflammatory Effect Is Mediated by Inhibition of DSS-Induced Adhesion Molecules in the Colon

**Figure 5:**
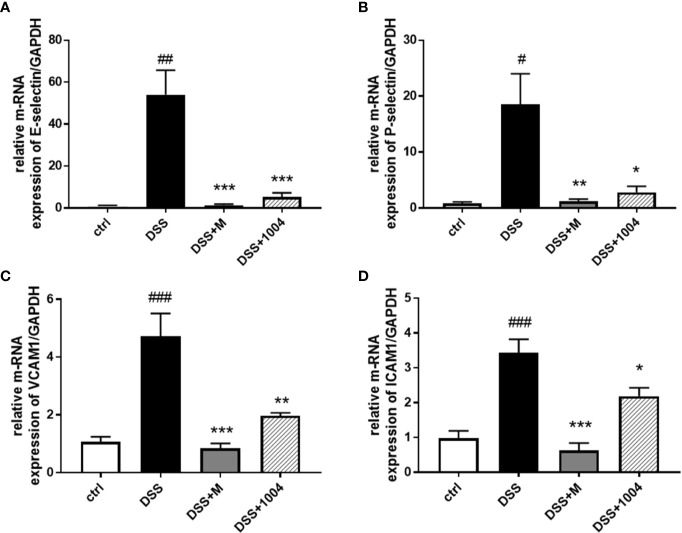
Downregulation of adhesion molecule expression in CU06-1004-treated mice. Quantitative real-time polymerase chain reaction (RT-PCR) was performed using gut homogenates to detect adhesion molecule expression levels. E-selectin **(A)**, P-selectin **(B)**, VCAM-1 **(C)**, and ICAM-1 **(D)**. ^#^P < 0.05, ^##^P < 0.01, ^##^P < 0.01, versus the control group. *P < 0.05, **P < 0.01, **P < 0.01, ***P < 0.001 versus the dextran sodium sulfate (DSS) group. (n=4–5 per group).

Leukocyte rolling, adhesion, and transendothelial migration from the bloodstream into the tissues of the colon are critical steps in the development of the IBD inflammatory response. In the DSS group, pronounced increases in colonic E-selectin (**Figure 5A**), P-selectin (**Figure 5B**), VCAM-1 (**Figure 5C**), and ICAM-1(**Figure 5D**) expressions were seen. However, the upregulation of RNA for these adhesion molecules was markedly inhibited in CU06-1004-treated mice (**Figure 5**). Therefore, the reduced inflammation in CU06-1004-treated colonic tissue (**Figure 2B**) is likely mediated by a decrease in leukocyte transendothelial infiltration of the colon *via* downregulation of adhesion molecules (**Figure 5**). Because mesalazine is an antiinflammatory and immunosuppressive drug, the expression of adhesion molecules in mesalazine-treated mice decreased even more than in the CU06-1004 treated groups, but it may cause severe side effects.

### CU06-1004 Attenuated Epithelial Inflammation and Loss of Epithelial Barrier Integrity

**Figure 6:**
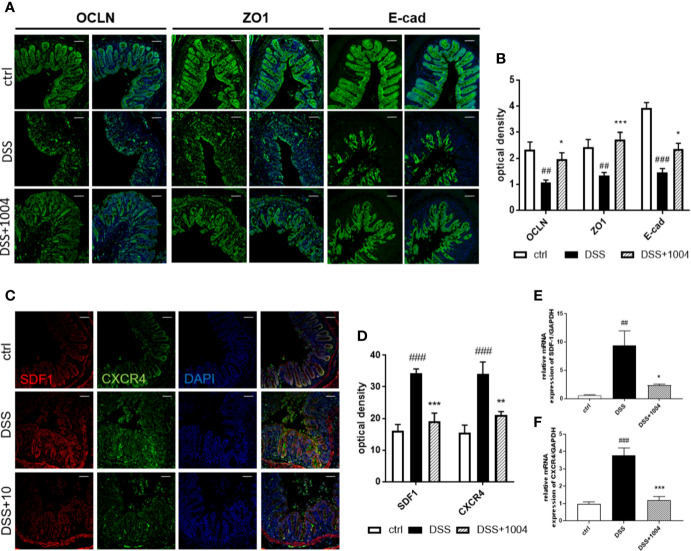
CU06-1004 reduced epithelial barrier destruction and epithelial inflammation. Epithelial junctions (occluding, Zonula occludens-1, and E-cadherin expressions) were upregulated in the CU06-1004-treated group. Magnification 200×, scale bar: 50 μm **(A)**. Epithelial junctions were quantified by optical density **(B)**. Levels of chemokine SDF-1 and its receptor CXCR4 were evaluated using immune-histochemistry **(C, D)** and RT-PCR on RNA from gut homogenates (n=4–5 per group) **(E, F)**. ^##^P < 0.01, ^###^P < 0.001 versus the control group. *P < 0.05, **P < 0.01, ***P < 0.001 versus the dextran sodium sulfate (DSS) group.

Destruction of the gut epithelium occurs afterward endothelium injury and contributes to IBD clinical manifestation (Tolstanova et al., 2012). In the early phase of DSS-induced colitis (days 2-3) there was no damage to the colonic epithelium (**Figures S4D** and **S5D** in **Supplementary Material**), but the vessels did lose their junctions (**Figures S4G–J** and **S5 G–J** in **Supplementary Material**). This suggests that vessel destruction precedes epithelial injury. Indeed, inhibition of vascular dysfunction (e.g. through treatment with CU06-1004) prevents epithelial injury (as observed from immunohistochemistry), which relieved clinical symptoms. Epithelial tight junctions and adherens junctions showed strong expression in the control-group mice, but DSS-group mice showed weakened expression. However, colon samples from CU06-1004-treated mice showed more regular and stronger expression (**Figures 6A, B**), indicating restoration of epithelial barrier integrity with CU06-1004 treatment. SDF-1, secreted by damaged colonic epithelial cells, facilitates leukocyte infiltration, and increases severity during the development of UC (Dotan et al., 2010). Both SDF-1 and its main receptor, CXCR4, are known to increase during UC development. Colonic expression of both SDF-1 and CXCR4 derived by epithelium (**Figures 6C, D**) and decreased in CU06-1004-treated mice compared with DSS mice (**Figures 6C–F**). Therefore, CU06-1004 induces vessel protection, which inhibits epithelial damage and has a therapeutic benefit in colitis.

## Discussion

Various factors can cause IBD, including environmental, genetic, and lifestyle factors, and infection (Vedamurthy and Ananthakrishnan, 2019). Many studies have investigated IBD pathogenesis, but its exact etiology remains unclear. Colonic blood vessel dysfunction is one factor that often influences the development of IBD (Cromer et al., 2011). Therefore, blood vessel protection is a promising strategy to block IBD development. The DSS mouse model often used in the process of drug discovery because of its ease of use and high success rate for disease induction. DSS administration causes colonic inflammation, ulceration, crypt dilation, edema, and mixed immune cell infiltration — all similar characteristics of UC (Blumberg et al., 1999). Here we report that CU06-1004 has a tissue-protection effect on DSS-induced UC by attenuating endothelial dysfunction and inflammation.

The gut changes induced by DSS occurred in an early phase (days 2–3) and a late phase (days 4–5). In the early phase, DSS injured endothelial cells in the deep layer of the lamina propria directly or indirectly and induced endothelial dysfunction. Endothelial dysfunction characterized by reduced barrier function, upregulation of cellular adhesion molecules, and increased leukocyte diapedesis (Saijo et al., 2015; Sun et al., 2018). Colon blood vessels become twisted, dilated, and irregularly distributed, and vessel diameters reduced (Chidlow et al., 2007). Our results show that CU06-1004 increased junction protein expression levels, which, in turn, blocked DSS-induced increases in vascular permeability, and prevented further development of UC. Furthermore, immunostaining of colon whole mounts showed strengthened and less tortuous colonic blood vessels in CU06-1004-treated mice. CU06-1004 had reported protecting the endothelium from the junction-destroying influence of multiple cytokines, so we hypothesized that CU06-1004 would also prevent cytokine vasodilation effects (many studies have shown cytokine level increases in IBD (Cromer et al., 2011)), even though it did not reduce some cytokine level directly (data not shown).

Increased expression of adhesion molecules is another characteristic of endothelial dysfunction. It leads to abnormal leukocyte extravasation from the blood into colonic tissue, contributes to the devastation of the epithelium, and plays a crucial role in the pathogenesis of colitis. Leukocytes infiltrate the region in three steps; rolling, activation, and adhesion. During leukocyte-endothelium interactions, various adhesive and migratory molecular events occur, with selectin and cell adhesion molecules (CAMs) having the most relevance. The initial process for leukocyte recruitment is selectin-mediated low-affinity and transient tethering and rolling of leukocytes, followed by integrin-dependent firm adhesion (Ley et al., 2007). Our results indicate that CU06-1004 significantly suppressed selectin and CAM expression, and reduced leukocyte extravasation by preventing both the rolling and the adhesion process. Therefore, reduced adhesion and leukocyte infiltration could feedback into the inflammatory process and attenuate it. A reduction in the inflammatory response, seen as a decrease in the proinflammatory cytokines IL-1β, IL-6, and TNF-α, also observed in the CU06-1004-treated mice intestine. These cytokines amplify inflammatory cascades and result in intestinal tissue damage in UC patients. The treatment of UC often involves the downregulation of TNF-α using antibodies (e.g., infliximab and adalimumab) (Argollo and Danese, 2019). IL-1β is known to stimulate diarrhea, immune cell infiltration, and intestinal necrosis, and IL-6 reduction had shown to slow down the development of UC and colitis-associated colon cancer (Papadakis and Targan, 2000; Perrier and Rutgeerts, 2011; Moriasi et al., 2012; Song et al., 2013). Although an antibody-based drug can target its corresponding cytokine, CU06-1004 inhibits the effects of multiple cytokines. Therefore, the overall effect of CU06-1004 is to reduce inflammation by reducing the expression of endothelial adhesion molecules and inhibiting multiple cytokines.

In the late phase of UC, the destruction of the mucosal epithelium occurs and leads to crypt pathology disorders (Saijo et al., 2015). As a result of vascular protection, this late-phase feature of epithelial injury also decreased in CU06-1004-treated mice. Improved epithelial integrity and decreased epithelial inflammation was verified by assessing junction protein expression and SDF-1 expression (reported to be upregulated in injured epithelium, particularly in colitis (Zimmerman et al., 2008; Dotan et al., 2010; Kulkarni et al., 2017)). Reduced epithelial injury also reduced the primary clinical manifestations of fecal blood, weight loss, and loose stools. These three features were verified each day and represented by the DAI. Upon analysis of the colon at the early time points, after mice had been administered DSS for 3 days, there were no significant changes in colonic epithelium, but the mice presented with vascular junction loss and the beginnings of inflammatory cell infiltration due to increased vascular permeability. However, CU06-1004 treated mice had stronger junction protein expression and decreased immune cell infiltration than the DSS-alone group (**Figures S5D–J** in **Supplementary Material**). Therefore, CU06-1004 induces vessel protection before epithelial damage can occur, providing therapeutic benefits to protect against colitis. This is an important insight into the mechanism by which CU06-1004 modulates colonic blood vessels and further protects colonic tissue.

Due to its unknown etiology, there is no precise treatment for IBD, and the application of most drugs depends on disease severity (timing of the disease) and specific location. Typical treatments usually consist of supportive medications and immunosuppressive drugs, which have many undesirable side effects and complications (Wilhelm and Bryan, 2017; Seyedian et al., 2019). Therefore, the development of better and different therapeutic approaches that have practical effects and minimal or no side effects is very important. CU06-1004 treatment after 7 days of DSS administration led to milder disease severity, as reflected by improvements in body weight (increased) and diarrhea (less severe) relative to the DSS group (**Figure S6** in **Supplementary Material**). Further, the colonic vessel integrity was increased in the CU06-1004 mice relative to the DSS-treated group (**Figure S7** in **Supplementary Material**). This may suggested that CU06-1004 also has therapeutic effects with protective effects on against colitis by improving vascular integrity. Moreover, since CU06-1004 regulates vessels, and not the immune system directly, there may be fewer side effects than other immunomodulatory drugs. Consequently, CU06-1004 may be beneficial for the treatment of IBD both during and after disease progression in safe.

In conclusion, the results demonstrate that oral administration of CU06-1004 has a therapeutic influence on DSS-induced UC. CU06-1004 treatment reduced vascular permeability by upregulating junction protein expression, reduced leukocyte extravasation by downregulating adhesion molecule expression, decreased proinflammatory cytokine expression, and increased vessel normality in the colon. Furthermore, epithelial integrity, which has a pivotal role in colitis, was improved indirectly following reduced endothelial dysfunction. Moreover, CU06-1004 not only has protective effects, but also clinical effects, aiding the recovery of severely damaged colon tissue. These findings suggest that colonic vessel integrity is vital to colitis inhibition and recovery. Therefore, the endothelial dysfunction blocker CU06-1004 may provide a novel protective and treatment strategy for the pharmacological treatment of IBD. And this suggests an important insight into therapeutic access of IBD, not directly impede the immune system.

## Data Availability Statement

The raw data supporting the conclusions of this article will be made available by the authors, without undue reservation, to any qualified researcher.

## Ethics Statement

The studies involving animals were reviewed and approved by the Institutional Animal Care and Use Committee of Yonsei University, Seoul, Korea.

## Author Contributions

Y-SK and HZ and carried out the experimental work. Y-SK wrote the manuscript. HZ proofread the manuscript. SL contributed revision experiment. MN and SP contributed data analysis. Y-MK and Y-GK supervised and edited the manuscript. All authors contributed to the article and approved the submitted version.

## Funding

This work was supported by the Basic Science Research Program through the National Research Foundation of Korea funded by the Ministry of Education, Science and Technology [grant number 2019R1A2C3007142], and the Bio & Medical Technology Development Program of the National Research Foundation of Korea [NRF-2015M3A9B6066835].

## Conflict of Interest

Author HZ was employed by the company Curacle Co. Ltd.

The remaining authors declare that the research was conducted in the absence of any commercial or financial relationships that could be construed as a potential conflict of interest.
