# Supplementary material for: CU06-1004 Alleviates Experimental Colitis by Modulating Colonic Vessel Dysfunction
Source: Front Pharmacol. 2020 Sep 15;11:571266. doi: 10.3389/fphar.2020.571266 (PMC7523507; doi:10.3389/fphar.2020.571266)
Supplement: Supplementary file 1 [file DataSheet_1.docx]

Supplementary Material


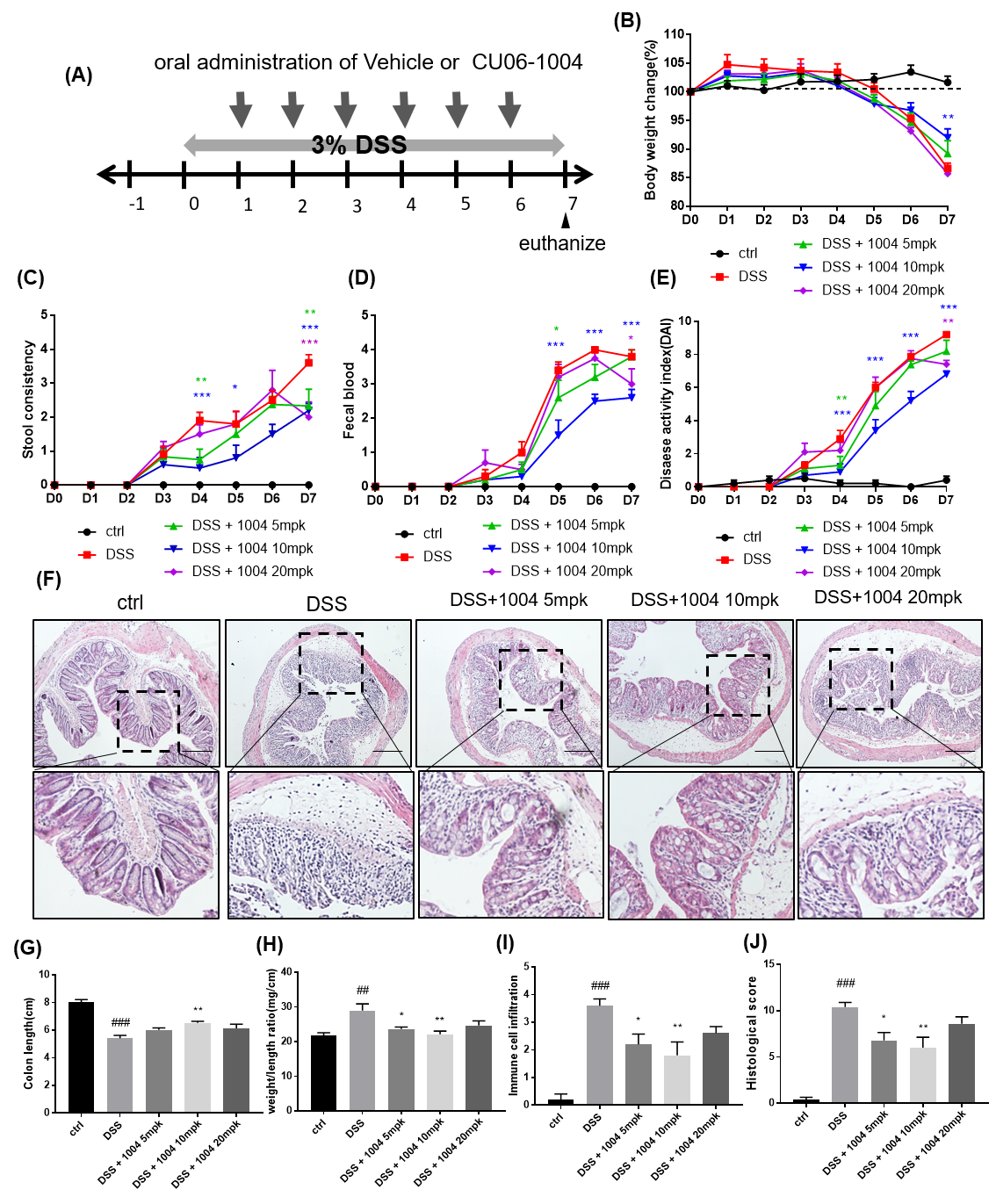


**Supplementary Figure 1.** **CU06-1004 at a dose of 10 mg/kg provides the strongest therapeutic effect.**

Mice were divided into five groups; control (ctrl.), vehicle (Dextran Sodium Sulfate, DSS), and 3 concentrations of CU06-1004 (DSS+1004 5/10/ 20 mg/kg [mpk]). The control (normal) group received water and the other groups received DSS. Mice were administered 3% DSS *ad libitum* for seven days and treated with vehicle or CU06-1004 24 hours after DSS administration started. CU06-1004 was given at 5, 10, 20 mg/kg, for six days (A). Body-weight change (%) (B), diarrhea (C), and fecal blood scores (D) were determined each day, and their sums determined a disease activity index (DAI) score (E). Mice were sacrificed and colons were obtained seven days after DSS administration. The colon length was measured for length (to represent extent of edema) (G), and weight-length ratios (H), and then sectioned and stained with H&E. Magnification 100×, scale bar: 20 μm (F). Histopathological scores were then assessed (I-J). ##P < 0.01, ###P < 0.001 versus the control group. *P < 0.05, **P < 0.01, ***P < 0.001 versus the DSS group. (n=5 per group)


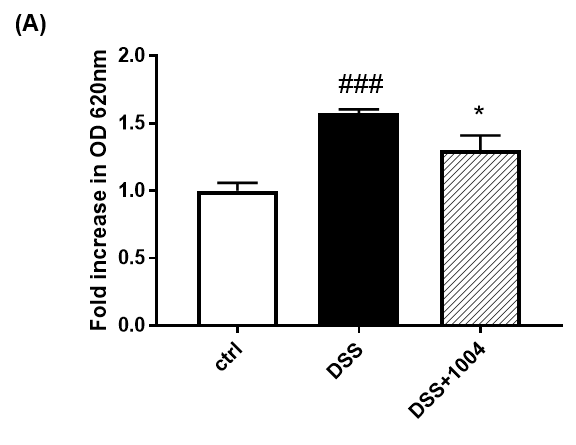


**Supplementary Figure 2. CU06-1004 reduces colonic vascular permeability**

Colonic vascular permeability was measured using Evan’s blue assay. The extent of dye leakage increased in the DSS-administered group but was reduced in the CU06-1004 treated mice relative to the DSS group. ###P < 0.001 versus the control group. *P < 0.05 versus the DSS group. (n=6 per group)


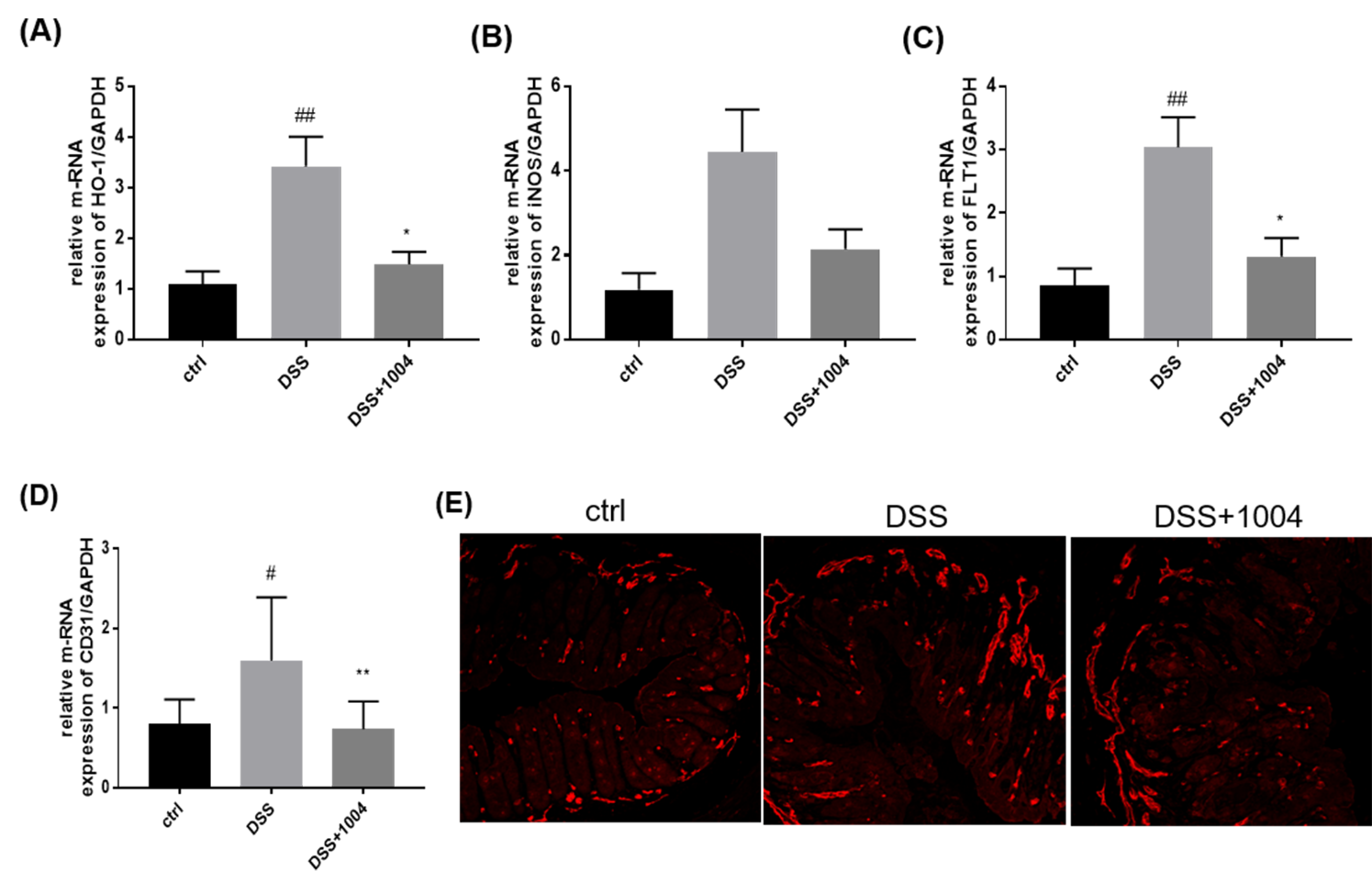


**Supplementary Figure 3. CU06-1004 reduces hypoxia and pathological angiogenesis.**

qRT-PCR was performed using gut homogenates to detect HO-1 (A), iNOS (B), FLT1(C), and CD31 (D). CD31 IHC staining of colon (E). #P < 0.05, ##P < 0.01 versus the control group. *P < 0.05, **P < 0.01 versus the DSS group. (n=4-5 per group)


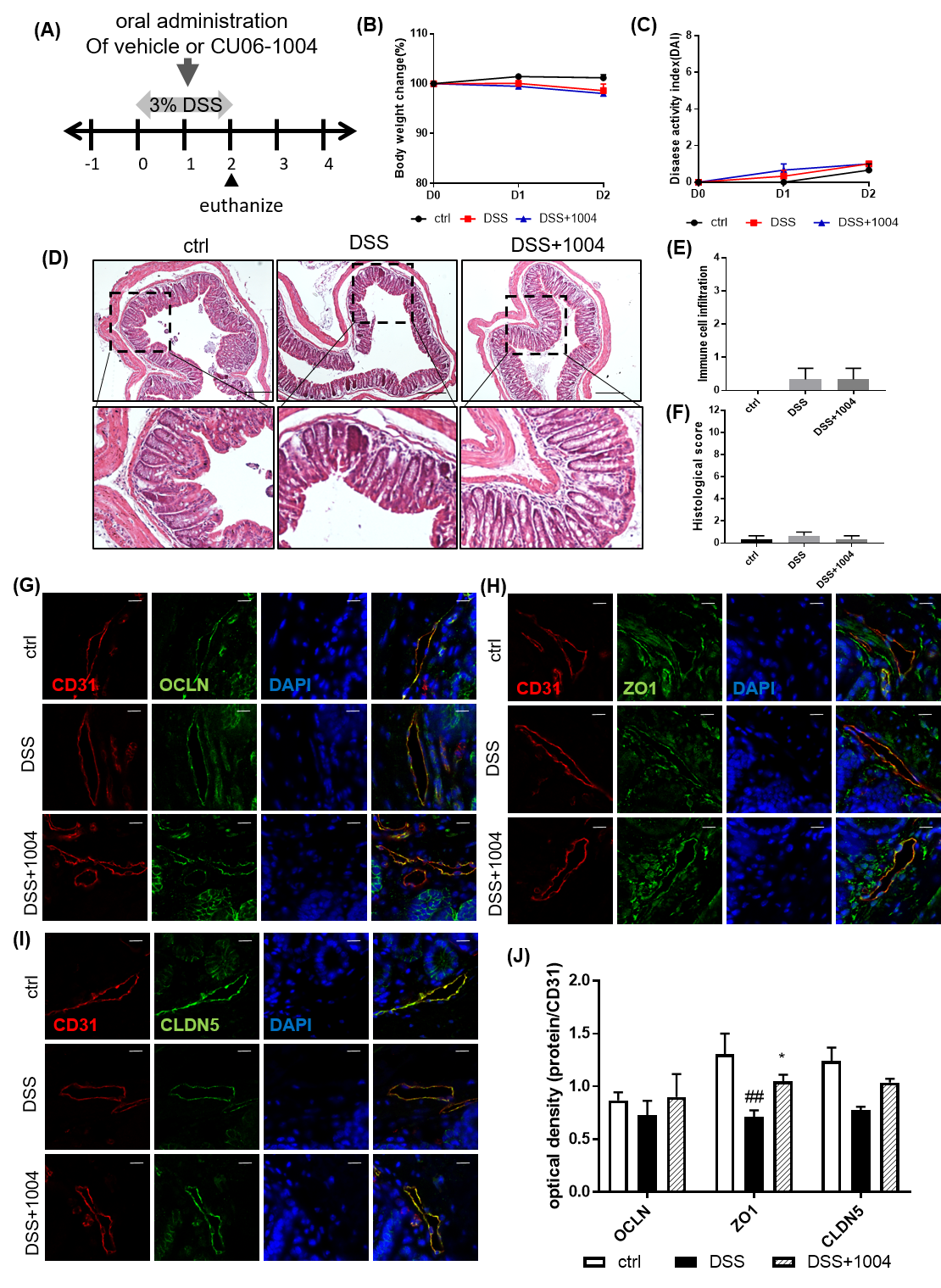


**Supplementary Figure 4. CU06-1004 inhibits endothelial dysfunction before epithelium injury and clinical manifestations; colon analysis in the early phase (day 2) of DSS-induced colitis**.

Mice were divided into three groups; control (ctrl.), vehicle (DSS), and CU06-1004. The control (normal) group received water, and the other groups received DSS. Mice were administered 3% DSS *ad libitum* for two days and were treated with the vehicle or CU06-1004 24 hours after initiation of DSS administration. CU06-1004 was administered at 10 mg/kg for one day (A). Body-weight change (%) (B), diarrhea, and fecal blood scores (data not shown) were determined each day, and their sums determined a disease activity index (DAI) score (C). Colons were obtained two days after DSS administration and then sectioned and stained with H&E. Magnification 100×, scale bar: 20 μm (D). Histopathological scores were then assessed (E-F). Immunofluorescence staining of colon sections were assessed for CD31 (red) and the tight junction proteins (green) occludin (G), Zonula occludens-1 (H), and claudin-5 (I). Sections were counterstained with DAPI (blue nuclei). CD31 and tight junction co-expression was quantified by optical density (J). The co-expression of junction proteins and vascular markers represents the colon's vascular barrier. Magnification 1,000×, scale bar: 10 μm, ##P < 0.01 versus the control group. *P < 0.05 versus the DSS group (n=3 per group).


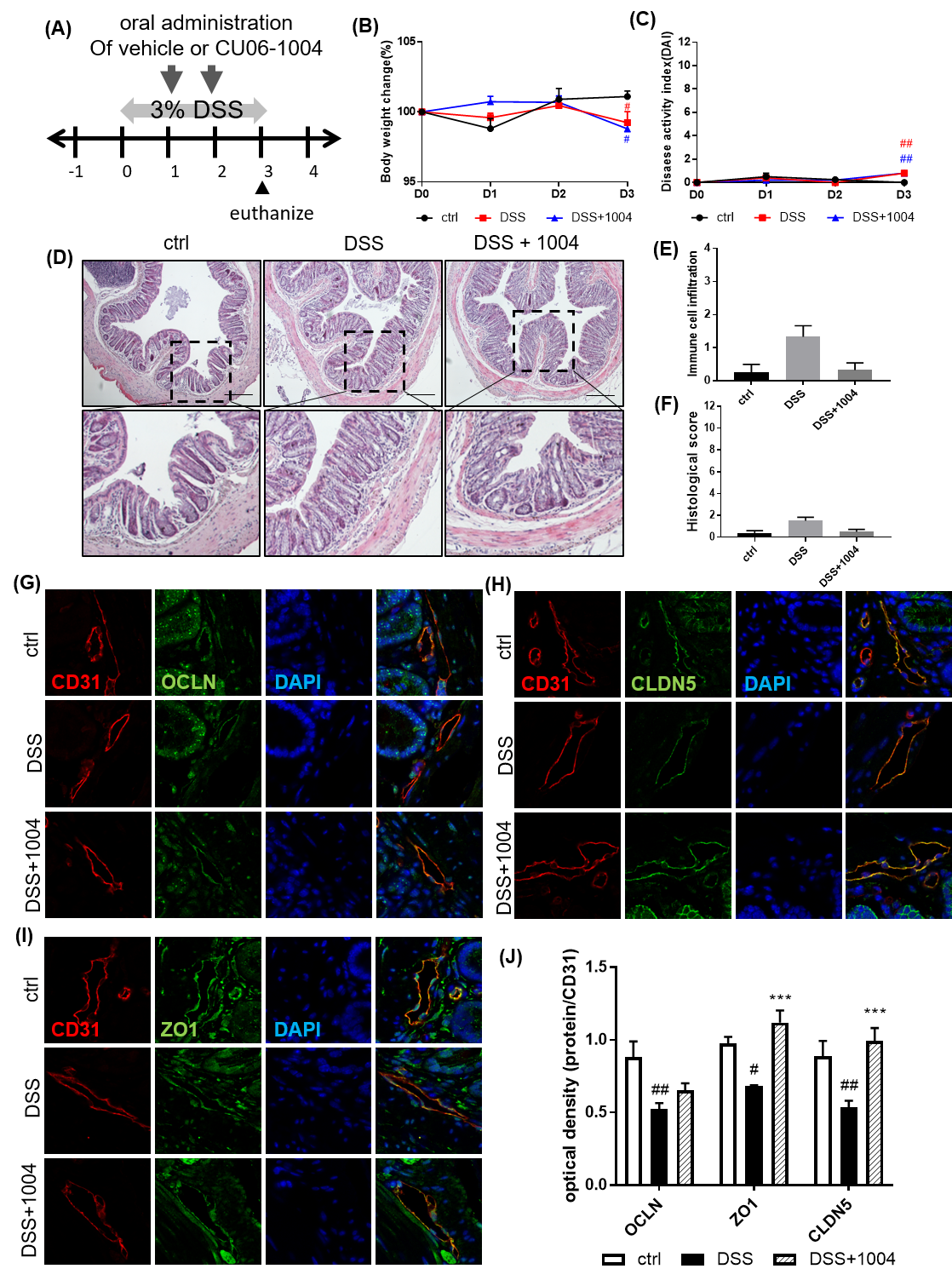


**Supplementary Figure 5. CU06-1004 inhibits endothelial dysfunction before epithelium injury and clinical manifestation; colon analysis in the early phase (day 3) of DSS-induced colitis.**

Mice were divided into three groups; control (ctrl.), vehicle (DSS), and CU06-1004. The control (normal) group received water, and the other groups received DSS. Mice were administered 3% DSS *ad libitum* for three days and were treated with vehicle or CU06-1004 24 hours after that initiation of DSS administration. CU06-1004 was administered at a dose of 10 mg/kg for two days (A). Body-weight change (%) (B), diarrhea, and fecal blood scores (data not shown) were determined each day, and their sums determined a disease activity index (DAI) score (C). Colons were obtained two days after DSS administration and then sectioned and stained with H&E. Magnification 100×, scale bar: 20 μm (D). Histopathological scores were then assessed (E-F). Immunofluorescence staining of colon sections were assessed for CD31 (red) and the tight junction proteins (green) occludin (G), Zonula occludens-1 (H), and claudin-5 (I). Sections were counterstained with DAPI (blue nuclei). CD31 and tight junction co-expression was quantified by optical density (J). The co-expression of junction proteins and vascular markers represents the colon's vascular barrier. Magnification 1,000×, scale bar: 10 μm, ##P < 0.01 versus the control group. #P < 0.05, ##P < 0.01 versus the control group. ***P < 0.001 versus the DSS group (n=4-5 per group).


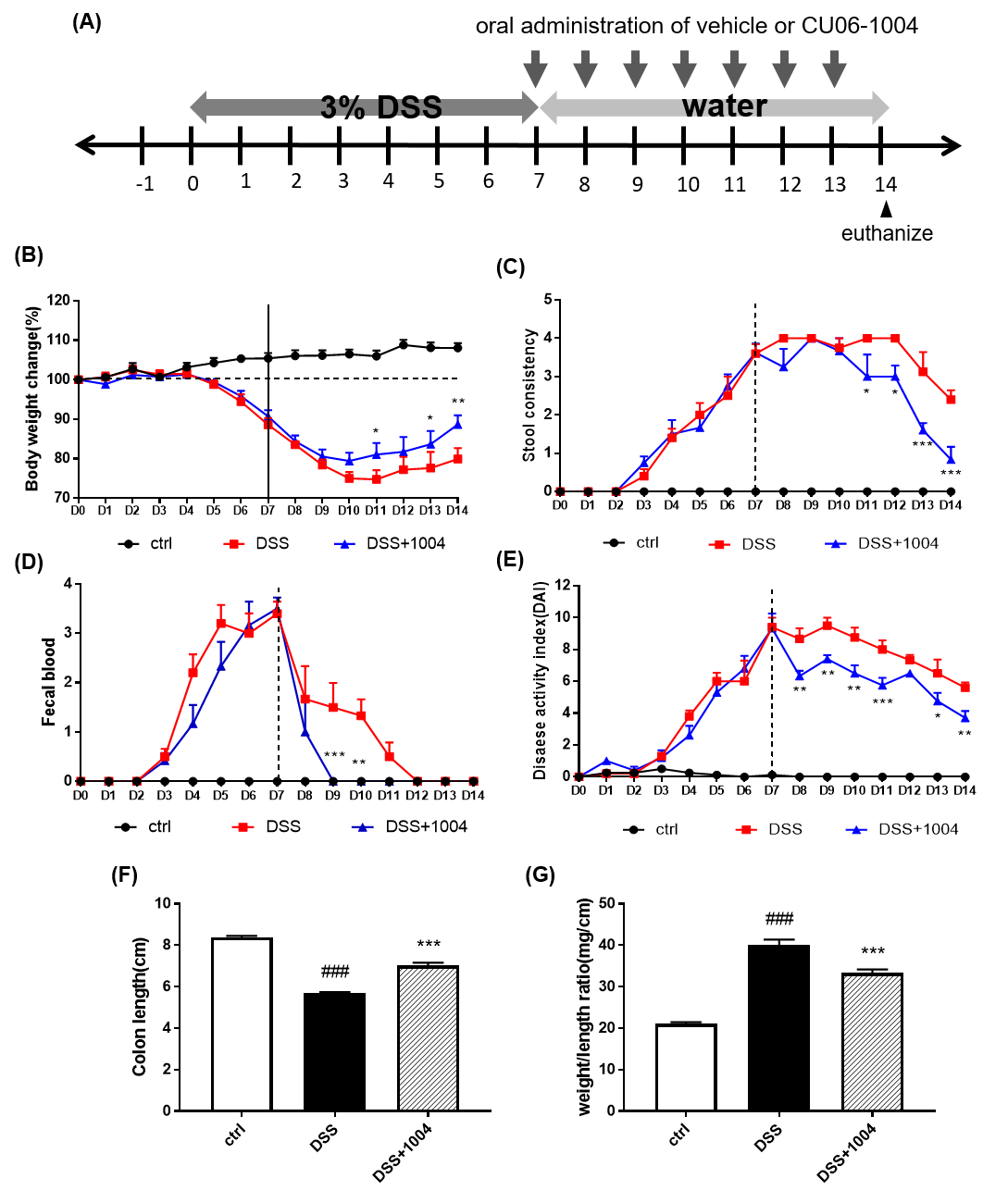


**Supplementary Figure 6. CU06-1004 attenuates DSS-induced colitis after disease progression.**

Mice were divided into three groups; control (ctrl.), vehicle (DSS), and CU06-1004. The control (normal) group received water, and the other groups received 3% DSS for seven days. After day 7, all groups of mice received water (with no DSS). Mouse were treated with vehicle or 10 mg/kg CU06-1004 on day 7 through day 13 (A). Body-weight change (%) (B), diarrhea (C), and fecal blood (D) scores were determined each day, and their sums determined a disease activity index (DAI) score (E). Colons were obtained fourteen days after DSS administration and measured for colon length (F) and weight-length ratio (edema rate). ###P < 0.001 versus the control group. *P < 0.05, **P < 0.01, ***P < 0.001 versus the DSS group. (n=5 per group).


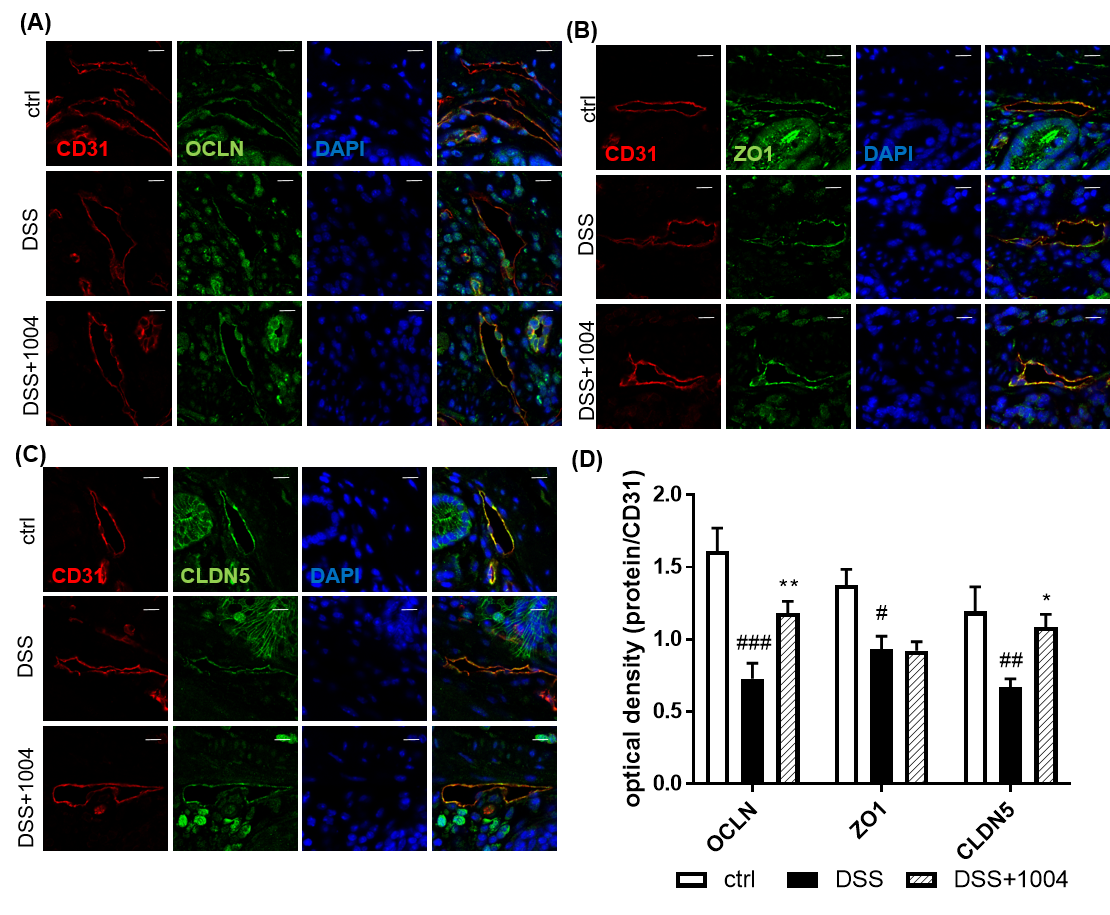


**Supplementary Figure 7. CU06-1004 improves colonic vascular integrity.**

Immunofluorescence staining of colon sections for CD31 (red) and the tight junction proteins (green) occludin (A), Zonula occludens-1 (B), and claudin-5 (C). Sections were counterstained with DAPI (blue nuclei). CD31 and tight junction co-expression was quantified by optical density (D). The co-expression of junction proteins and vascular markers represents the colon's vascular barrier. Magnification 1,000×, scale bar: 10 μm, #P < 0.05, ##P < 0.01, ###P < 0.001 versus the control group. *P < 0.05, **P < 0.01 versus the DSS group. (n=5 per group)
